# Safety and Therapeutic Profile of a GnRH-Based Vaccine Candidate Directed to Prostate Cancer. A 10-Year Follow-Up of Patients Vaccinated With Heberprovac

**DOI:** 10.3389/fonc.2019.00049

**Published:** 2019-02-25

**Authors:** Jesús A. Junco, Ranfis Rodríguez, Franklin Fuentes, Idania Baladrón, Maria D. Castro, Lesvia Calzada, Carmen Valenzuela, Eddy Bover, Eulogio Pimentel, Roberto Basulto, Niurka Arteaga, Angel Cid-Arregui, Francisco Sariol, Lourdes González, Liliana Porres-Fong, María Medina, Ayni Rodríguez, A. Hilda Garay, Osvaldo Reyes, Matilde López, Lourdes de Quesada, Allelin Alvarez, Carolina Martínez, Marleny Marrero, Guillermo Molero, Alfredo Guerra, Pedro Rosales, Carlos Capote, Sahily Acosta, Idania Vela, Lina Arzuaga, Ana Campal, Erlán Ruiz, Elier Rubio, Pável Cedeño, María Carmen Sánchez, Pedro Cardoso, Rolando Morán, Yairis Fernández, Magalys Campos, Henio Touduri, Dania Bacardi, Indalecio Feria, Amilcar Ramirez, Karelia Cosme, Pedro López Saura, Maricel Quintana, Verena Muzio, Ricardo Bringas, Marta Ayala, Mario Mendoza, Luis E. Fernández, Adriana Carr, Luis Herrera, Gerardo Guillén

**Affiliations:** ^1^Center for Genetic Engineering and Biotechnology of Camaguey, Camagüey, Cuba; ^2^Uro-oncology Department of National Institute of Oncology and Radiobiology (INOR), Havana, Cuba; ^3^Center for Genetic Engineering and Biotechnology, Havana, Cuba; ^4^Center for Molecular Immunology, Havana, Cuba; ^5^German Cancer Research Center, Heidelberg, Germany; ^6^Oncologic Hospital of Camaguey, Marie Curie, Camagüey, Cuba; ^7^Department of Pharmacology of Camaguey Medical University, Camagüey, Cuba; ^8^Amalia Simoni Clinical-Surgical Hospital, Camagüey, Cuba; ^9^Clinical Laboratory of the Oncologic Hospital of Camaguey, Marie Curie, Camagüey, Cuba; ^10^Clinical Trials Department of Oncologic Hospital Marie Curie of Camaguey, Marie Curie, Camagüey, Cuba; ^11^BioCubafarma, Havana, Cuba

**Keywords:** advanced prostate cancer, GnRH/LHRH vaccine, hormone ablation, hormone sensitive cancer, overall survival

## Abstract

Heberprovac is a GnRH based vaccine candidate containing 2.4 mg of the GnRHm1-TT peptide as the main active principle; 245 μg of the very small size proteoliposomes adjuvant (VSSP); and 350 μL of Montanide ISA 51 VG oil adjuvant. The aim of this study was to assess the safety and tolerance of the Heberprovac in advanced prostate cancer patients as well as its capacity to induce anti-GnRH antibodies, the subsequent effects on serum levels of testosterone and PSA and the patient overall survival. The study included eight patients with histologically-proven advanced prostate cancer with indication for hormonal therapy, who received seven intramuscular immunizations with Heberprovac within 18 weeks. Anti-GnRH antibody titers, testosterone and PSA levels, as well as clinical parameters were recorded and evaluated. The vaccine was well tolerated. Significant reductions in serum levels of testosterone and PSA were seen after four immunizations. Castrate levels of testosterone were observed in all patients at the end of the immunization schedule, which remained at the lowest level for at least 20 months. In a 10-year follow-up three out of six patients who completed the entire trial survived. In contrast only one out eight patients survived in the same period in a matched randomly selected group receiving standard anti-hormonal treatment. Heberprovac vaccination showed a good security profile, as well as immunological, biochemical and, most importantly, clinical benefit. The vaccinated group displayed survival advantage compared with the reference group that received standard treatment. These results warrant further clinical trials with Heberprovac involving a larger cohort.

## Introduction

The early landmark studies of Huggins and Hodges established the hormonal dependence of prostate cancer and provided the basis for the use of androgen deprivation in its treatment (1).

Reduction of plasma testosterone to castrate levels, either through surgical castration (orchiectomy), or of oral or injectable estrogens, became the standard therapy for disseminated prostate cancer in the following 40 years (2–6). In the early 1980s, LHRH analogs were added as an alternative to achieve reversible pharmacologic castration (7–10).

By the mid 1990's, an immunological approach (LHRH vaccines) had been designed and tested in men to achieve androgen deprivation to treat prostate cancer (11, 12) and in post-menopausal women to test gonadotropin inhibition (13). The efficacy of the neutralizing action of LHRH/GnRH through the involvement of hormone-specific antibodies has been demonstrated in a wide range of animal species, including humans. Such studies have involved either passive immunization by infusion of anti-LHRH antibodies (14) or vaccination with the LHRH peptide coupled to tetanus or Diphtheria toxoid (DT) molecules as carriers (11–14), or LHRH in multiple antigen peptide (MAP) constructs (15). These approaches are impractical for widespread commercial application since passive immunity is inefficient and expensive (16) and the use of peptide–toxoid conjugates and MAP constructs produce variable results (17). On top of that, the GnRH-tetanic toxoid conjugates since their big size can induce anti-haptenic immunosuppression and such process became difficult to reproduce at industrial scale (18).

In order to overcome these limitations, the Heberprovac vaccine candidate was designed, which contains the modified pEHWSYPLRPG GnRH sequence, chemically coupled to the 830–844 T helper epitope of the tetanic toxoid (TT) in the same synthetic process. Such approach breaks immune tolerance to hormone, by eliciting anti-LHRH neutralizing antibodies that induce immunological castration (19). The administration of seven Heberprovac immunizations, followed by radiotherapy in six advanced prostate cancer patients, resulted in 100% immunogenicity, testosterone drop to castration levels, and PSA normalization. These clinical results had never been reported for a GnRH-based vaccine.

## Materials and Methods

### Ethics and Methodological Aspects

The current clinical trial complied with the principles of the Declaration of Helsinki on clinical investigation in humans. It was approved by the Scientific and Ethics Committee of the Marie Curie Oncology Hospital, in Camaguey, Cuba, as well as by the National Regulatory Authority of Cuba (CECMED). The patient's informed consent was recorded before the study was started. An intermediate endpoint was established to identify the high-risk cases and poorly responding patients, who then received the usual disease treatment as recommended by the medical guidelines. The intermediate evaluation was setup to ensure protection of patients with low immunization response. The adverse events were evaluated by The Common Terminology Criteria for Adverse Events, Version 3.0^7^ http://ctep.cancer.gov/protocoldevelopment/electronic_applications/docs/ctcaev3.pdf.

### Trial Design

It was a single arm, open, prospective study in which a randomized external group of patients with locally advanced and metastatic prostate cancer was used. The main goal of the trial was to evaluate the product safety according to the local and systemic adverse events (AE) and signs of efficacy. The sample size (N) was calculated in 6–8 patients for the immunized and for the external group receiving the standard therapy. During the study, safety and tolerance of the vaccine candidate were monitored by rigorous control of the adverse events, and calculation of the occurrence frequency. The survival of vaccinated patients was compared with a cohort of patients bearing advanced prostate cancer, selected with the same criteria, received the standard anti-hormonal treatment.

### Patients and Eligibility

**Diagram 1:**
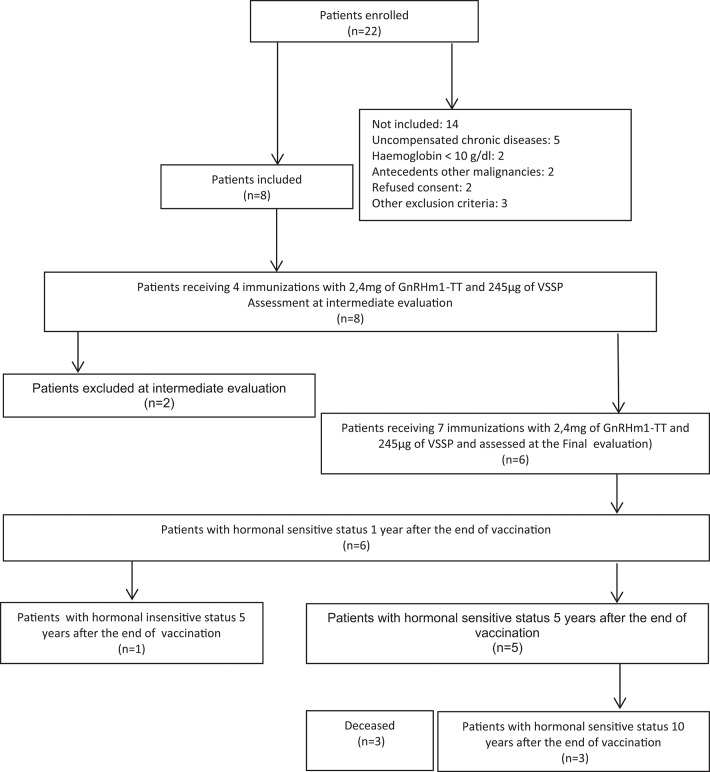
Outcome of patients included in the Phase I clinical trial with Heberprovac. CONSORT diagram.

From January to March 2007, eight men diagnosed with advanced (stage 3–4) prostate cancer (TNM classification, 1992) were recruited at the Uro-Oncology Department of the Marie Curie Oncology Hospital in Camaguey, Cuba, based on clinical, biochemical and anatomical-pathological criteria. Previously, all patients signed an informed consent. The prostate biopsy was performed using trans-rectal ultrasound with a biopsy device (ALOKA 2004, Japan). The eligibility criteria also included leukocytes >3.0 × 10^9^/L, lymphocytes >1 × 10^9^/L, thrombocytes >100 × 10^9^/L, and hematocrit >30%. The exclusion criteria for the treatment included previous immunological treatment of up to 2 months before the beginning of the immunization schedule, as well as significant levels of anti-GnRH antibodies, and decompensated chronic diseases (asthma, epilepsy, autoimmune diseases, immunodeficiency, anemia, uncontrolled urinary sepsis and renal, hepatic and cardiovascular diseases) Diagram 1.

### Vaccine Composition and Treatment Schedule

**Figure 1:**
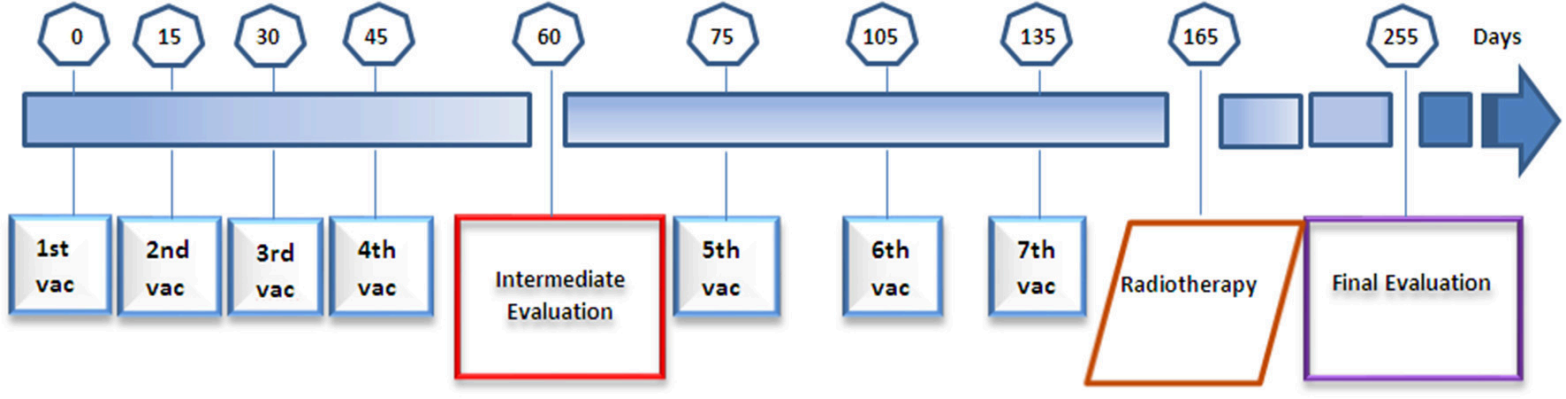
Schematic overview of the immunization schedule, radiotherapy, and evaluation interventions practiced to the patients participating in the Heberprovac Phase I clinical trial.

The vaccine consist of a mixture of three components: the 27 amino acid GnRHm1-TT peptide synthetized and supplied by The Center of Genetic Engineering and Biotechnology (CIGB), Cuba, in 2.4 mg 2R vials; Montanide ISA 51 VG adjuvant from Seppic, France; and VSSP, a Neisseria meningitidis derived adjuvant produced and supplied by the Center of Molecular Immunology (CIM), Cuba, in 0.8 mg/0.5 mL vials. Before immunization, the peptide was resuspended in VSSP adjuvant and mixed (50:50 v/v) with the Montanide ISA 51VG oil adjuvant, in order to form a water-in-oil emulsion that was added to a total volume of 700 μL, and injected intramuscularly to patients. All patients received seven doses of a vaccine containing 2.4 mg of the peptide, 245 μg of VSSP, and 500 μL of Montanide ISA-51. The first four doses were administered fortnightly, and the remaining three were applied monthly. A month after vaccination ended, a total of 60 Gy radiotherapy (RT) was assessed using a Co-60 radioisotope (Figure 1). The patients' response to vaccination was evaluated at recruitment, after the fourth and seventh immunizations, and after receiving RT.

### Clinical and Complementary Assessment

The patients underwent general physical examination, digital rectal exam (DRE), and laboratory imaging and analysis. The imagenological examination included transrectal and trans-abdominal ultrasound and bone gammagraphy to determine possible metastases. Blood samples were drawn for routine checkups at recruitment and 15 days after the fourth and seventh immunizations, and after the patients received RT for general clinical laboratory parameters, as well as for anti GnRH antibody titers, using an ELISA kit. For the biochemical and endocrine evaluation, serum PSA was determined by ultra-micro-analysis system (UMELISA, CIE, Cuba) and the testosterone levels were quantified through a radioimmune assay (RIA, CISBIO, France). Since the main goal of the trial was to measure the product safety, the local and systemic adverse events were carefully assessed. Systemic toxicity was evaluated for 72 h after each vaccination. It included measurements of temperature, blood pressure, respiratory frequency 30 min after each injection, and later, every hour during 4 h. The patients completed the physical examination in 72 h, using the standard supervision applied to in-patients, through anti-GnRH quantification plus serum PSA and testosterone determinations.

### Long Term Follow-Up of Patients

Follow up was made every 3–4 months for 10 years since the end of the immunization. The parameters evaluated in each medical consultation were the same as for the previous evaluation of patients during the clinical trial development: DRE, anti-GnRH antibodies, serum testosterone and PSA. Imaging methods: Trans-rectal ultrasound (Aloka, Japan) was used at the diagnosis and at the final evaluation for prostate biopsy. In order to look for nodules and metastases, we carried out Tc 99 Gammagraphy scan. After the completion of treatment the patients were followed up for a further period of 10 years. Survival of patients that completed the vaccination schedule was compared with a parallel sample of patients (*n* = 8) with similar disease status, who received standard anti-hormonal treatment.

### Statistics

The data were double entered and validated using Microsoft Access, and then imported into SPSS 13.0, for analysis. The frequency distribution and central tendency and dispersion were estimated by mean standard deviation, median, interquartilic range (QR), and the maximum and minimum values (range) for qualitative and quantitative variables.

For each type of adverse event, the frequency distribution (IC 95%) was estimated with the classical and Bayesian statistics. For survival, statistical analysis was carried out using Log Rank test.

## Results

### Study Population

Between March and July 2007, eight men with confirmed diagnosis of advanced prostate cancer (stages III/IV) were included in the safety study with the vaccine candidate Heberprovac. At the same time, 8 patients with advanced prostate cancer were randomly selected in the uro-oncology service, who began treatment with the standard therapy for prostate cancer and were used as external control group (EG). Tables 1A, B. The age of patients ranged from 63 to 78 years old (71.3 years on average). All patients had high Gleason score confirmed by the histological study. The patients were evaluated at recruitment, after the fourth and last (7th) immunizations, the later after they received the RT (Figure 1). The treatment schedule was completed in 6 patients, who were followed up for recurrence during 10 years (2007–2017) Diagram 1.

**Table 1A:** TNM classification and Gleason score of patients included in the Phase I clinical trial with Heberprovac.

**Prostate cancer patients vaccinated with Heberprovac**		
**Patient no**.	**TNM classification**	**Gleason score**
MC01	**T3M0N0: Stage III**. Prostatic tumor with extracapsular extension to both prostate lobules. No meta, no ganglionar nodules	Prostatic ADC Gleason 9 in all the studied fragments. Predominant pattern 4
MC02	**T3bM0N0: Stage III**. Prostatic tumor with extracapsular extension to both prostate lobules. No meta, no ganglionar nodules	Prostatic ADC Gleason 9 in left lobule. Gleason 8. Predominant pattern 4. Right Lobule hyperplastic
MC03	**T3bN0M1b Stage IV**. Prostatic tumor with extracapsular extension to both prostate lobules. Bone metastases. No ganglionar extension	Prostatic ADC Gleason 8 in 100 % of the simple. Right lobule Hyperplasia
MC04	**T4aN0M1b Stage IV**. Prostatic tumor with extracapsular extension to both prostate lobules that infiltrate vesical neck, rectus; with bone meta. No ganglionar infiltration	Prostatic ADC. Gleason 10 in all the studied fragment of the right and left lobules
MC05	**T3aN0M0**. **Stage III**. Prostatic tumor with extracapsular extension to one prostate lobule. No meta, no ganglionar nodules	Prostatic ADC of all studied fragment of right lobule. Gleason 8. Left lobule, ADC, Gleason 8 in 100% of the samples
MC06	**T3bN0M1b Stage IV**. Prostatic tumor with extracapsular extension to both prostate lobules. Bone metastases. No ganglionar extension	Undifferentiated Carcinoma of muscle tissue. Gleason 10
MC07	**T3aN0M0 Stage III**. Prostatic tumor with extracapsular extension to one prostate lobule. No meta, no ganglionar nodules	Prostatic ADC of right lobule. Gleason 8 in all the samples. Left lobule Hyperplastic
MC08	**T3aN0M0 T Stage III**. Prostatic tumor with extracapsular extension to one prostate lobule. No meta, no ganglionar nodules.	Prostatic ADC of left lobule. Gleason 10 in 100% of samples. Right lobule hyperplasic. Gleason 6

*TNM correspond to patient classification according to the American Joint Committee on Cancer (20)*.

**Table 1B:** TNM classification and Gleason score of patients non-included in the clinical trial that were used as control external group.

**Non included Prostate cancer patients (External group)**		
**Patient no**.	**TNM classification**	**Gleason score**
EG03	**T4 N1M0: Stage IV**. Prostatic tumor with extracapsular extension to both prostate lobules. No meta, ganglionar nodules	Prostatic ADC Gleason 8 in all (4) studied fragments. Predominant pattern 4
EG05	**T4 N0M1: Stage IV** prostatic tumor with extracapsular extension to both prostate lobules. Bone metastases, no nodules	Prostatic ADC Gleason 9 in both lobules. Predominant pattern 5
EG06	**T3aN0M0. Stage III**. Prostate tumor with perineural and perivascular extension to both prostate lobules. No Bone metastases. No ganglionar extension	Prostatic ADC Gleason 7 in 4 out 5 samples studied. Predominant pattern 4
EG09	**T4b N1M1b Stage IV**. prostatic tumor with extracapsular extension to both prostate lobules that infiltrate bladder. Bone metastases and ganglionar infiltration	Prostatic ADC. Gleason 10 in all the studied fragments of the right and left lobules
EG11	**T3bN0M0**. **Stage III**. Prostatic tumor with extracapsular extension to both prostate lobules. No metastases, no ganglionar infliltration	Prostatic ADC of the prostate. Right lobe, Gleason 8. Left lobe, Gleason 9 in all the samples
EG12	**T3bN1M1b Stage IV**. Prostatic tumor with extracapsular extension to both prostate lobules. Bone metastases. No ganglionar extension	Undifferentiated Carcinoma of prostate with muscle tissue infiltration. Gleason 10
EG14	**T3aN0M0 Stage III**. Prostatic tumor with extracapsular extension to one prostate lobule. No meta, no ganglionar nodules	Prostatic ADC of right lobule. Gleason 8 in all the samples. Predominant pattern 4
EG17	**T3aN0M0 T Stage III**. Prostatic tumor with extracapsular extension to one prostate lobule. No metastases	Prostatic ADC of right lobule. Gleason 9. Predominant pattern 5

*TNM correspond to patient classification according to the American Joint Committee on Cancer (20)*.

### Adverse Events

The vaccine was well tolerated despite the presence of side effects and adverse reactions (see below) that coincided with the protocol safety hypothesis. No vaccine-related events exceeded grade II. The intermediate evaluation was made to check safety. Two patients (04 and 06) were removed from the study for presenting signs of clinical and biochemical progression of the disease (interruption criteria).

The observed local and systemic adverse events are summarized in the Table 2. All patients reported local pain at the vaccination site. Three of them developed a slight swelling around the injection site. Other events reported were local redness and swelling, skin atrophy, induration, and erythema. Systemic adverse events included fever, muscle pain and flu-like symptoms in all the six patients that finished the treatment. Late adverse events were mainly associated with the hormone deprivation caused by the vaccine, and included libido decrease, sexual dysfunction, breast tenderness and weakness. Remarkably, not a single case of Gynecomastia was observed for the vaccinated group. However, in the case of the control group, it is important to point out that 75% of patients reported hot flushes between 15 and 20 days after the injection of Zoladex, as well as an increase in urinary symptoms after the first two administrations of the GnRH analog. Similarly, symptoms depending of hormonal ablation as asthenia, sexual erectile dysfunction and decreased libido were observed in the 60–100% of patients, respectively (Table 2).

**Table 2:** Most reported adverse events in Heberprovac vaccinated and control group prostate cancer patients.

**Variables registered by patients**		**Vaccinated**	**%**	**Control Group (%)**	
Local	Pain in the injection site	5	62.5	1	12.5
	Edema	5	62.5	1	12.5
	Skin atrophy	4	50.0	2	25.0
	Increase of volume	3	37.5	1	12.5
	Erythema	2	25.0	0	0.00
	Induration	4	50.0	0	0.00
	Crusty lesion	4	50.0	0	0.00
	Residual macula	1	12.5	0	0.00
	Scarring reaction	1	12.5	0	0.00
Systemic	Fever	6	75.0	1	12.5
	Anemia	1	12.5	3	37.5
	Asthenia	3	37.5	5	62.5
	Bradycardia	1	12.5	2	25.0
	Headache	1	12.5	2	25.0
	Depression	1	12.5	3	37.5
	Decreased libido	6	75.0	8	100.0
	Diarrhea	1	12.5	1	12.5
	Sexual erectile dysfunction	4	50.0	7	87.5
	Hypertension	3	37.5	2	25.0
	Hot flushes	0	12.5	6	75.0
	Gynecomastia	0	0.00	5	62.25

### Clinical and Imaging Evaluations

The evaluation of prostate size according to the DRE at recruitment for the trial showed that 7/8 patients possessed T3 prostate size, while one patient (MC04) displayed T4 prostate; the largest prostate size according to TNM classification (20). These data were confirmed using trans-rectal ultrasound.

Of the eight patients initially included in the trial, six completed the immunization schedule, and in two cases (patients MC04 and MC06) the treatment was interrupted and the patients had to abandon the trial after the intermediate evaluation, due to progression of the disease manifested as elevated PSA and creatinine, urinary obstruction, hydronephrosis, and renal failure that forced them to discontinue immunization.

The completion of treatment with the 7th Heberprovac immunization plus RT, resulted in a significant reduction of the prostate size in the six patients that concluded the full schedule and in the 100% of patients of the control group, considering the prostate size by DRE and trans-rectal ultrasonography.

**Figure 2:**
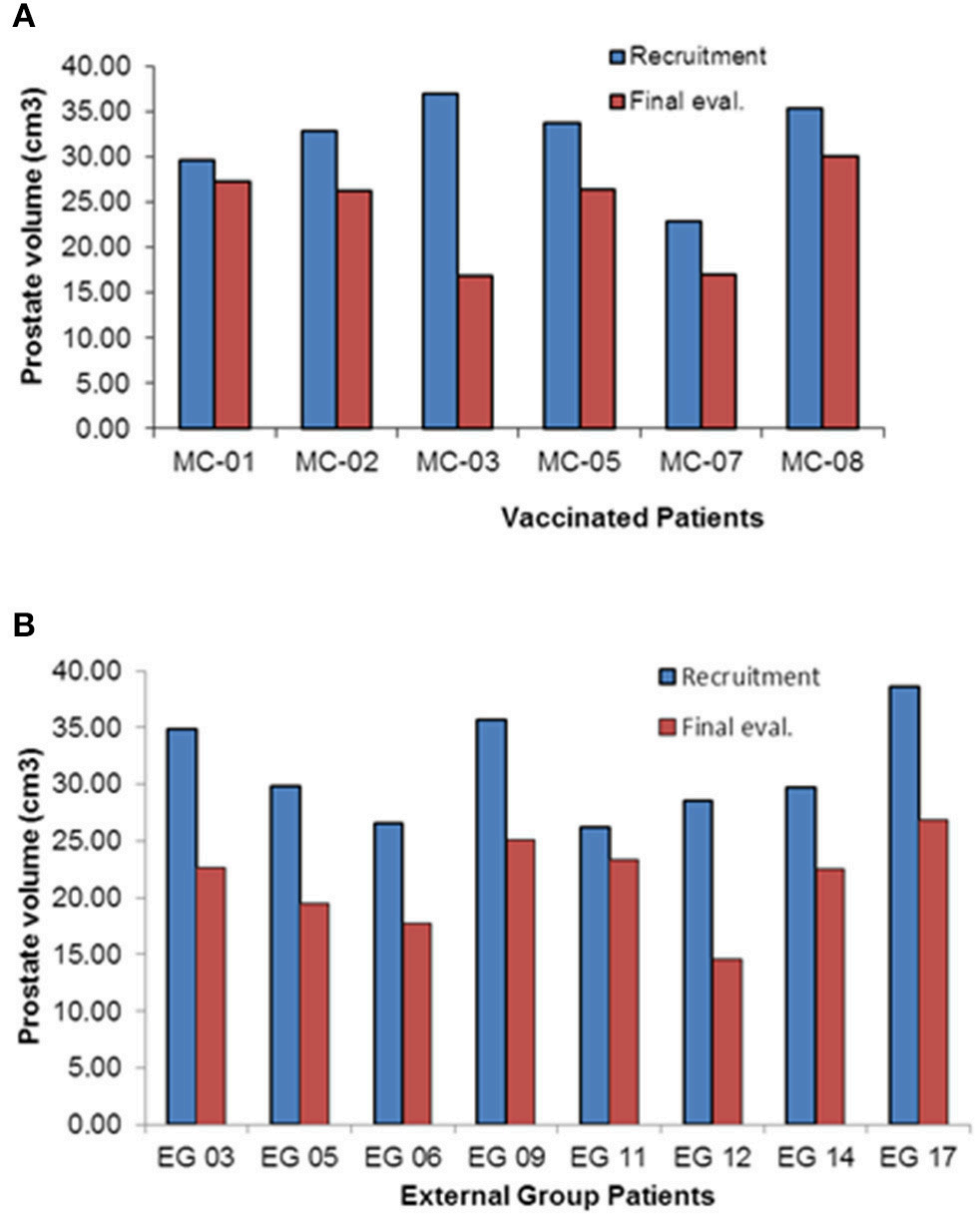
Prostate volume evaluation by trans-rectal ultrasound of the prostate cancer patients included in the clinical Heberprovac clinical trial and the External Group of prostate cancer patients with similar stage. **(A)** Individual measurement of prostate volume of patients before the treatment and after finishing the full immunization schedule and RT. **(B)** External Group prostate measurement using transrectal ultrasound before and after complete standard hormonal therapy and RT.

Transrectal ultrasound data of prostate volume for each patient is summarized in Figure 2. For immunized patients, the most important prostate reduction was observed in the patient MC 03, with 55% prostate reduction. Patients MC 07, MC 05, and MC 02 underwent between 20 and 25% prostate volume reduction, whereas patient MC 08 had around 18% reduction. Patient MC 01 just suffered a 5% of prostate reduction at the time Figure 2A. The overall prostate volume reduction observed was 23.4%, in comparison to the moment of recruitment (*P* < 0.01). On the other hand, patients who received standard therapy also had an important benefit in relation to the reduction of the size of the prostate. In this way patients EG 03, EG 05, EG 06, and EG17 had a decrease of 30% or more of the prostate size. The patient EG 12 was the one that showed a greater reduction of prostatic size among all with 49%. The remaining 2 patients showed a decrease of 10 and 29% of the prostatic volume in relation with the beginning (Figure 2B).

### Anti-GnRH Immune Response and Surrogate Biochemical Markers

Heberprovac is a vaccine candidate designed to generate anti-GnRH antibodies. Such humoral immune response was evaluated at the mid and end stages of the trial and compared with the values at the moment of patient recruitment.

Table 3 shows Testosterone and PSA correspondence with the anti GnRH antibody titers. All patients generated anti-GnRH antibodies after the fourth immunization. Two patients (MC 03 and MC 05) developed 1:6,400 anti-GnRH antibody titers; three patients (MC01, MC 02, and MC 07) reached 1:3,200; two patients (MC 04 and MC 06) developed 1:1,600 titers; and one patient (MC 08), developed 1:800 anti-GnRH antibody titers. After completion of the reminder three immunizations, the anti-GnRH immune responses continued increasing and reached 1:25,600 in patient MC 05. Four patients (MC 01, MC 02, MC 03, MC 07) generated 1:12,800 antibody titers. The lowest anti-GnRH antibody response corresponded to patient MC 08, who developed 1:6,400 anti-GnRH titers. As mentioned previously, patients MC 04 and MC 06 showed disease progression, and did not complete the treatment; hence, they were excluded from the final evaluation.

**Table 3:** Anti GnRH antibodies, Testosterone and PSA levels at recruitment, intermedia and final evaluation of prostate cancer patients immunized with Heberprovac.

**Patient no**.	**Anti GnRH antibodies (Dilution titers)**		**Testosterone levels (nmol/L)**			**PSA levels (ng/ml)**		
	**After 4th immun**.	**After 7th immun**.	**At recruitment**	**After 4th immun**.	**After 7th immun**.	**At recruitment**	**After 4th immun**.	**After 7th immun**.
MC-01	3,200	12,800	4.4	0.13	0.249	22.9	12.49	0.43
MC-02	3,200	12,800	2.31	2.75	0.079	32.3	15.86	0.52
MC-03	6,400	12,800	4.55	2.04	0.041	46	25.50	0.83
MC-04	1,600	^*^	3.91	3.15	^*^	34.9	45.17^*^	^*^
MC-05	6,400	25,600	2.79	3.82	0.99	50	31.08	3.99
MC-06	1,600	^*^	4.94	4.68	^*^	16	22.95^*^	^*^
MC-07	3,200	12,800	3.39	6.46	0.10	3.80	2.09	0.36
MC-08	800	6,400	4.02	1.85	0.02	6.90	6.91	0.78

* *Means that the patient interrupted the treatment and were not evaluated at this time*.

Such anti-GnRH immune responses corresponded with a significant drop in testosterone, found in 3/8 patients (MC 01, MC 03, and MC 08) just 15 days after the fourth immunization. Upon completion of the immunization schedule and the conclusion of the radiotherapy, 100% of the patients that met the criteria of continuity in the trial, underwent testosterone castration under 1 nmol/l (*p* < 0.001) (Table 3).

**Figure 3:**
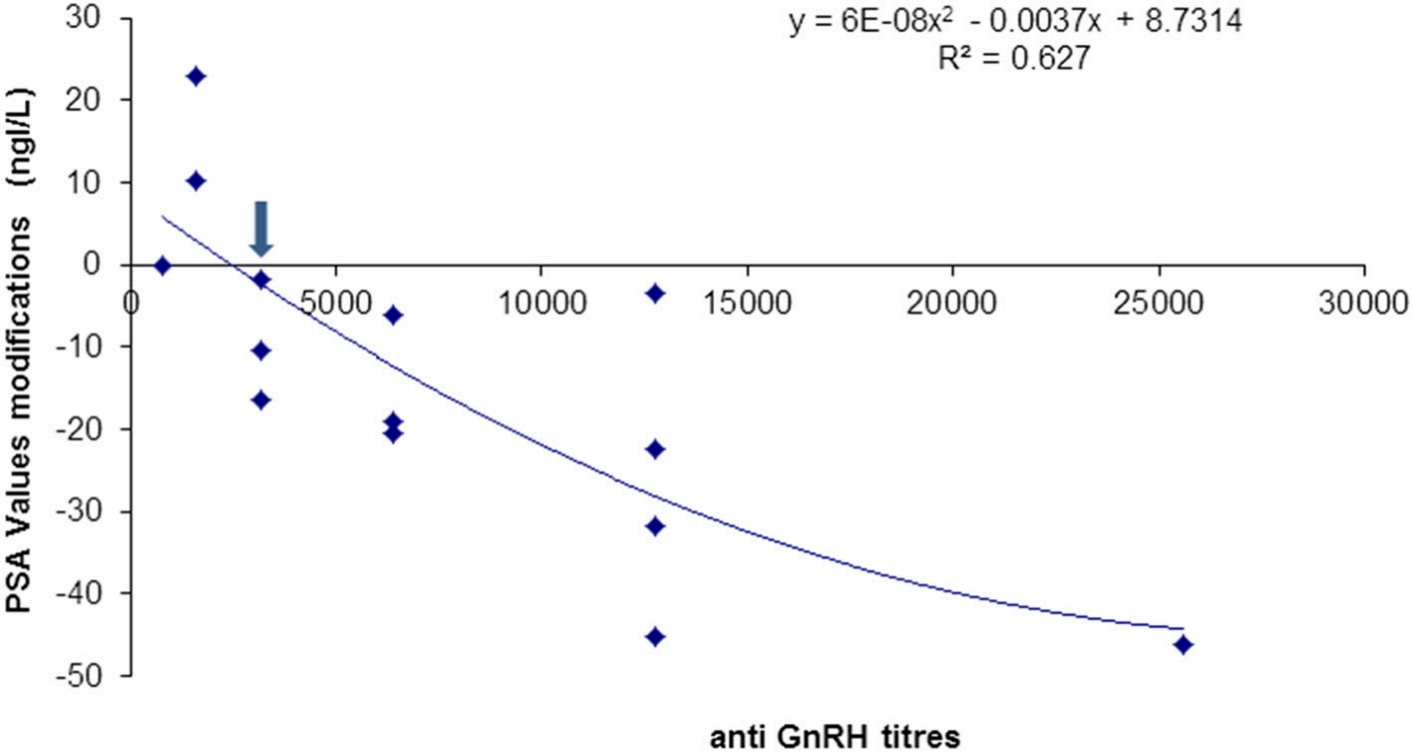
Anti-GnRH antibody titers and PSA values modifications in patients immunized with Heberprovac. Anti-GnRH antibody titers of 1:3,000 or higher (arrow), correlated with a decrease in the PSA values in all patients. A statistical correlation using a quadratic regression was significant (*R*^2^ = 0.627).

The patient's PSA kinetics was evaluated in parallel during the entire immunization schedule. Such measurements experienced a change from a mean of 26.6 ng/ml at recruitment, to 20.2 ng/ml after the fourth immunization (*p* > 0.05). The completion of the immunization schedule however, yielded complete PSA normalization in the six patients that concluded the protocol (*p* < 0.001) (Table 3). It is important to note that the PSA decline started when the anti-GnRH antibodies reached titers similar to or higher than 1:3,000. Figure 3 represents the inverse relation between anti-GnRH antibody titers and the PSA levels, the higher the anti GnRH titers, the lower the PSA values.

**Figure 4:**
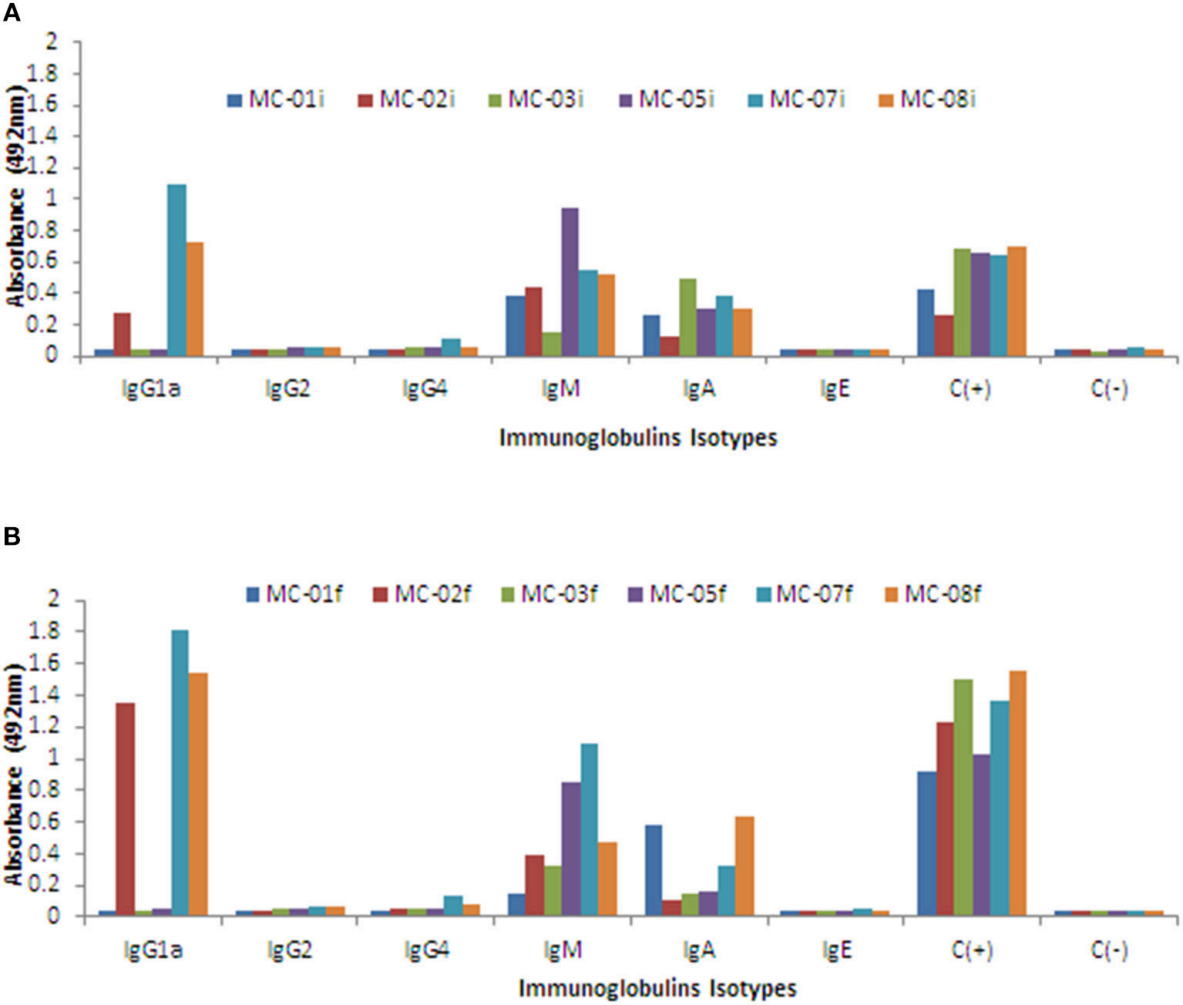
Schematic representation of anti GnRH antibody levels by isotypes tested during the intermediate and final evaluation of prostate cancer patients immunized with Heberprovac. **(A)** Individual values of anti-GnRH seroconversion by isotypes after the administration of 4 doses of Heberprovac. The most significant anti-GnRH antibody seroconversion were of IgM, IgG1, and IgA isotypes. **(B)** Individual anti GnRH seroconversion by isotypes of prostate cancer patents that completed all seven immunizations and received RT. The higher anti-GnRH antibody titers were found for IgG1, IgM, and IgA isotypes, respectively. Statistical significance was calculated using an ANOVA test followed by the Dunn comparison test. The i and f that appear in the legend of **(A,B)** refer to the intermediate and final evaluations, respectively.

Also, the anti-GnRH antibody isotypes generated with the vaccine candidate Heberprovac were determined. After finishing the fourth immunization, the highest antibody response in all the patients was of IgM subtype, followed by IgG1 and IgA, in that order (Figure 4A). After the end of the immunization schedule and once the patients had received the radiotherapy, the IgG1 isotype increased significantly and exceeded the IgM values. The IgM anti-GnRH immune-response however, kept a more regular distribution among all the patients that finished the trial. Besides, the IgG2, IgG4, and IgE in the serum samples represented < 10% of the total immunoglobulins detected (Figure 4B).

### Long Term Clinical, Biochemical, and Immunological Follow-Up of Patients During 10 Years

The primary endpoint of this phase I clinical trial of the vaccine candidate Heberprovac was to evaluate the acute and long term safety of the product which are described in 3.2 and Table 2.

#### Progression Free Survival Time (PFS) and Overall Survival (OS)

**Figure 5:**
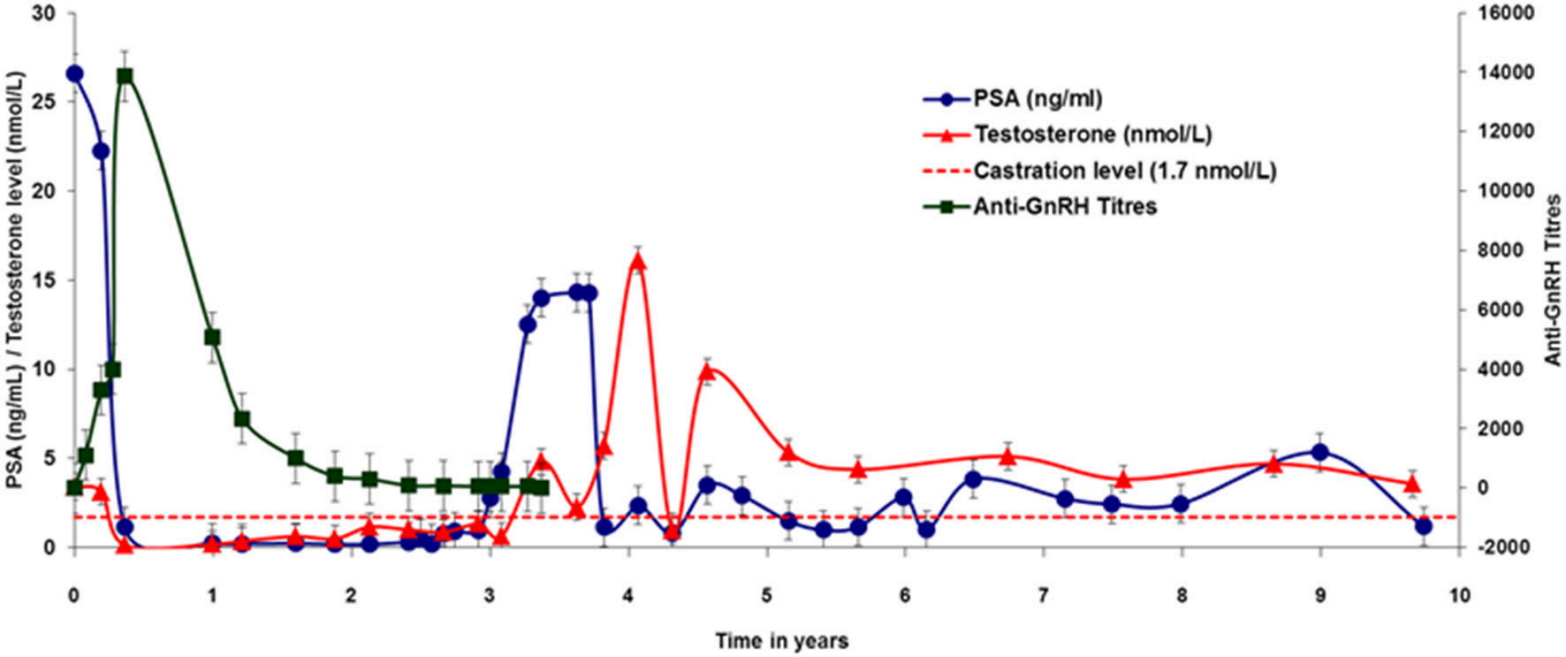
Ten-year follow up of 6 patients that completed the trial schedule with Heberprovac and received RT. The colored lines and each point represent the mean of the anti GnRH antibody titres (black), Testosterone values (nmol/L), (red), and PSA levels (ng/mL), (blue) at different moments of the trial. Maximal antibody titers corresponded with the Testosterone decrease to castration levels and PSA normalization between 5 and 6 months after the beginning of the trial. Note that, after an initial peak, the antibody titers dropped to about 50% 1 year after the treatment was completed, and were nearly cero at the end of the second year. However, testosterone continued at castration levels and PSA stayed normalized until the third year after treatment. Peaks of testosterone and PSA were observed between years 3 and 5 and corresponded to patients relapsed. Prism Graph Pad v6.1 was used for graph.

The secondary endpoint of this study was to test the capacity of Heberprovac to induce anti-GnRH antibodies, to reduce testosterone and PSA serum levels and, most importantly, to determine the patient overall survival. The Figure 5 shows a correlation between anti-GnRH antibody titers, testosterone and PSA levels of the six patients receiving seven doses of Heberprovac and radiotherapy after a 10 year follow up. The highest anti-GnRH antibody titers in serum were reached immediately after the end of the vaccination schedule, ~5 months after the beginning of the trial, with a mean value of 1:14,000.

Accordingly, testosterone values dropped to castration levels, and PSA normalization was observed in all patients at the time of final evaluation. The patient follow up showed that a year after the start of vaccination, the anti-GnRH antibody titers dropped to about half (average 1:6,000) of those seen by the end of the vaccination schedule. The anti-GnRH titers continued to decrease over time, but values remained above background for about 3 years (Figure 5). In accordance with the anti-GnRH seroconversion, during this period the testosterone concentration in serum remained at castration levels, and the PSA levels continued normal. Patients MC 03 and MC 05 showed testosterone and PSA relapsing, which was controlled with additional standard hormonal therapy. However, patient MC 03, responded only temporarily to the additional second line of hormonal ablation, and died 3 and a half years after finishing the treatment.

Also, from 3.5 to 5 years post-immunization, an increase in the testosterone levels was observed in patients MC 01, MC 07, and MC 08 (Figure 5). But it just raised the PSA values in patient MC 05, who responded very fast to the use of GnRH analog Zoladex

**Figure 6:**
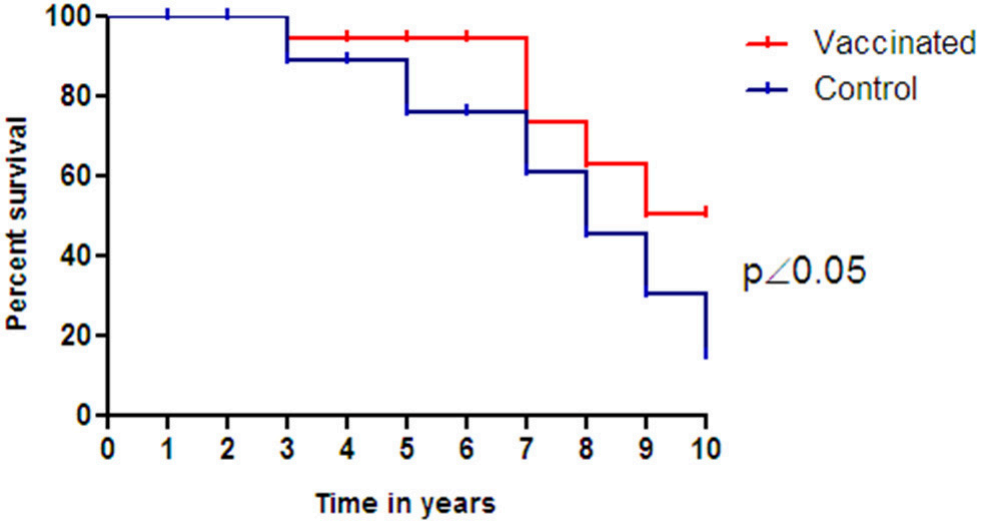
Kaplan-Meier curve for survival of prostate cancer patients receiving GnRH vaccination (Heberprovac) (*n* = 6) and patients that received standard anti-hormonal treatment during the same time period (*n* = 8). On completion of the treatment they were followed up for 10 years, after which 3 out 6 patients completing Heberprovac vaccination and 1 out 8 receiving the anti-hormonal standard treatment are alive. The statistical analysis was carried out using Log Rank test and demonstrated survival benefits for the vaccinated arm (*p* < 0.05).

The overall survival data of this study are summarized in the Figure 6. For the immunized group, patient MC 07, who maintained prostatic disease clinically and biochemically controlled, developed a primary lung cancer and died several months later. By the ninth year after the treatment, patient MC 08, who had never manifested PSA relapse or required any additional treatment for prostate cancer, died of pneumonia at age 82. Ten years after the end of the treatment with Heberprovac, 3 out of 6 patients that completed the treatment schedule are alive and have a clinically and biochemically controlled disease (Diagram 1). However, in the case of the control group that received standard anti-hormonal treatment, only 1 out 8 patients (12.5%) survive and keep hormone sensitiveness (Figure 6). The first patient of control group died after the third year (EG 09) as result of bone metastases and anemia. Patients EG 05 and EG 12 fell in a state of castration resistance and died at 5 and 7 years after the disease diagnosed, respectively. Patients EG 03 and EG 06 died from non-related prostate disease after 8 and 9 years of the treatment began, respectively. Finally, patient EG 11 suffered brain metastases and patient EG 14 was affected by bone metastases and kidney infiltration that generated renal insufficiency. Both patients succumbed 10 years after finished the treatment.

## Discussion

The combined use of adjuvant hormone and radiation therapies to treat high-risk prostate cancer patients has improved significantly results, with about 80% of patients disease-free (and no PSA failure) for 5 years (21).

The Gonadotropin-releasing hormone (GnRH) is critical for the normal functioning of the reproductive system. The administration of either polyvalent or monoclonal anti-GnRH antibodies in males, leads to decreased testicular size, cessation of spermatogenesis, and a severe reduction of testosterone levels, as does immunization with the GnRH-carrier conjugates (17, 22).

A number of studies have shown that the GnRH vaccines have promising application for managing hormone-dependent cancers (prostate and breast cancer) (23–25). However, the clinical application of these synthetic vaccines requires the availability of a powerful adjuvant to enhance antibody responses that effectively block hormone-receptor binding, for instance using GnRH analogs conjugated to bacterial toxoids, such as diphtheria (DT) or tetanic toxoid (TT) (26).

This paper describes a novel GnRH vaccine candidate (Heberprovac), which overcomes the limitations reported for other vaccine candidates in terms of anti-GnRH antibody responses and their efficacy. The fact that 100% of patients developed significant anti-GnRH antibody titers, and in turn all of them normalized or decreased PSA below 4 ng/mL during the final evaluation, represents an important achievement in relation to all the previous vaccine candidates based on GnRH (18, 27). Indeed, this is the first time that such efficient antibody responses have been reported using a GnRH-based vaccine.

The improved results provided by Heberprovac, could be partially considered as a consequence of amino acid change of L-Glycin by L-proline at the sixth position of the native GnRH that breaks the natural “U” conformation of the GnRH peptide. This change, along with the incorporation of the 830–844 TT epitope, leads to the formation of a longer and more rigid molecule that impairs hormone-receptor interaction and supports a better antigen processing and presentation thanks to its high promiscuity to existing haptenic molecules of different origins (12, 19, 28).

In addition, Heberprovac combines the GnRHm1-TT peptide formulated with the adjuvant Montanide ISA 51 (oil adjuvant) and VSSP, that belongs to the new generation of adjuvants based on pathogen-related molecules identified as danger signals recognized by the innate immune system (29). VSSP is proved to have the ability to activate mouse and human dendritic cells, *in vitro* and *in vivo*, with the corresponding IL-12p40/p70, TNF-α, and IL-6 production (30, 31).

Since Heberprovac effectiveness will depend mainly on the anti-hormonal effects caused by anti-GnRH antibodies capable of inducing immunocastration, the antibody titers, isotype maturity, and antibody affinity should correlate with such vaccine effects.

As expected, most anti-GnRH antibodies elicited after the first immunizations were of IgM isotype. At the end of immunization schedule, the antibodies switched to IgG1 and IgG2 subtype patterns in most patients. Several reports have shown that adjuvation of peptide vaccines with Montanide ISA 51 VG induces powerful antibody responses with a mixed Th1/Th2 profile, thanks to their capacity to expand lymphocyte subpopulations, particularly IFNγ that produces CD4 and CD8 T cells [production (30, 31)].

Regardless of the anti-GnRH antibody isotype proportion that prevailed in each patient, the testosterone values dropped significantly in all the cases at the end of the immunization schedule and radiotherapy. Interestingly, when the anti-GnRH antibody production reached titers ≥1:3,000, the PSA levels dropped to normal values in all the patients. This correlation could represent a prognostic indicator of patient responses to immunization with Heberprovac. However, further studies including a larger number of patients are required.

The high anti-GnRH immune response and the drastic reduction of testosterone levels in patients with advanced prostate cancer induced by Heberprovac in the current study, has not been reported before for similar candidates in clinical trials (11–13, 18, 28). Nevertheless, the most striking result of this study is, undoubtedly, the higher rate of survival after a 10-year follow up (see below). Remarkably, the immunological and endocrine parameters correlated with normalization of PSA serum levels in 100% of patients, elimination of urinary obstruction symptoms, and normalization of prostatic signs, according to the data obtained with the DRE and transrectal ultrasonography of the 6 patients who completed the clinical study. Interestingly, a year after the end of the trial, the breast tenderness observed during the first months disappeared, seemingly in relation to the discrete increase in the testosterone levels. A decrease in sexual libido was maintained while testosterone in serum remained at castration levels, and it was more evident in older patients (MC 02, MC 05, and MC 07). However, two of these patients had prior episodes of sexual erectile dysfunction. The remaining patients, including the MC 03 patient, who died of metastatic lesions 3 and a half years following treatment completion, showed a partial recovery of their sexual libido when the testosterone levels exceeded 5 nmol/L (data not shown). It was remarkable that, throughout the study, none of the patients suffered gynecomastia or hot flushes. However, the control group that received the standard antihormonal treatment, although it did not manifest any of the symptoms associated with the inflammatory response generated by the vaccines, showed a profuse symptomatology of testosterone suppression as the decrease in sexual libido, hot flushes, erectile sexual dysfunction and muscle weakness in the 60–100% of the patients, indistinctly. The occurrence of these adverse events, observed in the control group and commonly reported during hormonal therapy (32–35), were not observed with Heberprovac immunization. This is likely due to the gradual testosterone decrease induced by the vaccine in contrast to the rapid castration induced by analogs and antagonists of GnRH (36–40).

The long-term evaluation of patients immunized with Heberprovac, demonstrated a 50% survival in a 10 years follow up. In contrast, the parallel control group of patients receiving standard therapy for advanced prostate cancer demonstrated a significantly lower survival rate (12.5%) in the same period (*p* < 0.05) (Figure 6). We believe that the slow and progressive form of hormonal ablation produced by Heberprovac vaccination could be a determining factor in a longer delay in the transition from prostatic tumors to castration resistance (CRPC) and hence in the superior survival of Heberprovac vaccinated patients. Other aspects such as the value that the use of adjuvants such as VSSP could have in the generation of an immune spreading against the prostate tumor should also be explored.

Concerning long-term disease control in the vaccinated patients, only one patient (MC 03) died before 5 years of treatment. This case was a patient with metastatic prostate cancer at recruitment, and persistent symptoms of bone pain who, nevertheless, showed a vigorous immune response after vaccination that corresponded with a decrease in testosterone to castration levels, PSA normalization, and prostate size reduction, as shown by DRE and trans-rectal ultrasound. Besides this case, only one patient (MC 05) experienced a biochemical recurrence in the fourth year of the clinical trial and required hormonal treatment. Patients MC 01 and MC 08 showed a testosterone recovery of 10 and 15 nmol/L, respectively, however, they maintained normal levels of PSA, and did not require any additional treatment until 6.5 and 7 years.

Patients MC 07 and MC 02, both over 80 years old, died seven and 9 years after the start of the clinical trial, respectively, by causes unrelated to prostate cancer and its treatment. In both cases the patients exhibited complete disease control at the time of death, and never required additional hormone manipulation or another type of therapeutic strategy.

Altogether, these results are suggestive of a positive impact of vaccination with Heberprovac in overall patient survival compared with those receiving the standard treatment. Response to the vaccine correlated with the antibody titers raised against GnRH as well as with PSA reduction and castration levels of serum testosterone. Nevertheless, the value of such parameters as biomarkers of response need to be further confirmed in a future clinical trial with a larger cohort of prostate cancer patients.

## Author Contributions

JJ, RR, FF, IB, MDC, LC, CV, EB, EP, RBa, RBr, GG, AHG, AnC, AdC, ARa, AC-A, LH, and FS: conception and design of the study and writing and revision of the manuscript. LG, LP-F, ARo, AHG, OR, ML, MMed, LdQ, AA, CM, MMen, MMa, GM, AG, PR, RM, YF, MC, HT, DB, KC, PS, MQ, VM, MA, NA, CC, SA, IV, LA, ErR, ElR, PCe, PCa, MCS, IF, and LF: clinical trial assessment.

### Conflict of Interest Statement

The authors declare that the research was conducted in the absence of any commercial or financial relationships that could be construed as a potential conflict of interest.
